# Perceived Effectiveness and Utilization of Health Promotion Initiatives in Saudi Arabia: Insights and Recommendations

**DOI:** 10.3390/healthcare12232352

**Published:** 2024-11-25

**Authors:** Ebtihag O. Alenzi, Wasan Ibrahim Alqahtani, Milan Adeeb Altwegri, Sadeem Mobark Alhelal, Wadha Ahmad Alyami, Danah Mohana Almohana, Reem Rashed Aldrees, Rona Shagran Alnashar, Batool Hussain Almugizel, Noura Mohammed Alshabanat, Ghada Ali Alzahrani, Nouran Ehab Hassanein, Roaa Elkouny, Manal S. Fawzy

**Affiliations:** 1 Family and Community Medicine Department, College of Medicine, Princess Nourah bint Abdulrahman University, Riyadh 11671, Saudi Arabia; 2 Lifestyle and Health Research, Natural and Health Sciences Research Center, Princess Nourah bint Abdulrahman University, Riyadh 11564, Saudi Arabia; 3 College of Medicine, Princess Nourah bint Abdulrahman University, Riyadh 11671, Saudi Arabia; 441001248@pnu.edu.sa (W.I.A.); 441003702@pnu.edu.sa (M.A.A.); 441001266@pnu.edu.sa (S.M.A.); 441001629@pnu.edu.sa (W.A.A.); 439003304@pnu.edu.sa (D.M.A.); 441000184@pnu.edu.sa (R.R.A.); 441005121@pnu.edu.sa (R.S.A.); 441005720@pnu.edu.sa (B.H.A.); 439004499@pnu.edu.sa (N.M.A.); 441000329@pnu.edu.sa (G.A.A.); 4 College of Medicine, Alfaisal University, Riyadh 11533, Saudi Arabia; nouran2016@gmail.com (N.E.H.); roaaelkouny@gmail.com (R.E.); 5 Department of Biochemistry, Faculty of Medicine, Northern Border University, Arar 91431, Saudi Arabia; 6 Center for Health Research, Northern Border University, Arar 91431, Saudi Arabia

**Keywords:** healthy lifestyle, health promotions, initiatives, Saudi Arabia

## Abstract

**Background/Objectives:** Numerous national programs have been launched to enhance public health in Saudi Arabia, primarily aiming to promote healthy lifestyles through regular physical activity and a balanced diet. However, there is a lack of studies assessing the effectiveness and utilization of these initiatives. This study aimed to evaluate the perceived effectiveness and utilization of health promotion initiatives and identify the associated factors. **Methods:** A population-based cross-sectional study was conducted among adults (aged > 18 years) in Saudi Arabia using an online questionnaire through a convenient sampling approach. The questionnaire comprised three sections: sociodemographic data, medical history, and health promotion initiatives. An adjusted analysis was conducted using ordinary least squares (OLS) regression. **Results:** A total of 999 participants completed the survey. Walking paths emerged as the most perceived effective initiative, while labeled caloric menus were the most utilized. Perceptions of walking paths varied by age and health status; individuals aged 25–44 and lower-income groups viewed them less favorably. In contrast, those in good health found them more effective. Perceptions of calorie-related information on menus differed according to health status. Saudis perceived taxes on soft drinks less favorably than non-Saudis. Regionally, the western region favored fresh juice options compared to the East. The utilization of walking paths was higher among married individuals and those without chronic conditions, while the consumption of soft drinks was significant among younger and extremely obese individuals. **Conclusions:** The study revealed diverse perceptions and utilization patterns regarding health promotion initiatives among various demographic and socioeconomic groups, emphasizing the need for tailored strategies to enhance their effectiveness across populations.

## 1. Introduction

Health promotion initiatives are systematic efforts aimed at enabling and empowering individuals and communities to gain control over factors that influence their health and well-being. These initiatives achieve this by providing knowledge, resources, and support [1,2,3]. Typically, these efforts involve a range of strategies designed to promote healthy behaviors, prevent diseases, and enhance overall quality of life. By addressing risk factors, they encourage individuals to adopt healthier lifestyles, resulting in reduced morbidity and mortality rates [4,5]. Moreover, health promotion initiatives target various aspects of well-being, including physical, mental, and social health. They often require collaboration among multiple stakeholders, such as governments, healthcare organizations, non-governmental organizations (NGOs), and community groups [2].

In recent decades, there have been significant socioeconomic transformations in Saudi Arabia, leading to changes in lifestyle patterns, which could contribute to the increase in the prevalence of non-communicable diseases (NCDs), such as diabetes, obesity, and cardiovascular diseases [6,7]. Over the years, Saudi Arabia has implemented a range of health promotion programs aimed at mitigating the rising burden of NCDs. These initiatives signify the government’s commitment to public health amidst significant lifestyle changes attributed to urbanization and economic growth [8]. For instance, the “Healthy Mall” initiative not only encourages physical activity but also promotes community engagement in health-promoting behaviors [9]. Additionally, there are well-prepared paths for walking in public parks in all regions of the Kingdom [10]. In addition, the “Saudi Food and Drug Authority” mandated caloric labeling for all restaurants and coffee shops at the beginning of 2019, followed by increased awareness among the Saudi community about this calorie labeling law in restaurants and cafés menus [11,12]. Furthermore, in 2017, the Zakat, Tax and Customs Authority implemented an excise tax of 50% on beverages with added sugar and carbonation and 100% on energy drinks [13]. The excise tax has proven to dramatically impact people who rely on fizzy drinks for their daily caloric intake, even though it was unlikely to have a substantial short-term impact on the entire population [14]. In Saudi Arabia, the annual purchases (volume per capita) of soda and energy drinks decreased by 41% and 58%, respectively, in 2018 compared to 2016 [15]. Despite these efforts, the uptake of such initiatives remains variable across demographic groups, highlighting the need for targeted approaches that address specific community needs. Several programs have yielded promising results. The introduction of caloric labeling, for example, has raised awareness about dietary choices among consumers, potentially influencing purchasing habits [16]. However, the impact of such initiatives can be inconsistent. The excise tax on sugary beverages, while effective in reducing soda and energy drink purchases, has not fully addressed the broader nutrition and health challenges faced by the population [17]. Understanding the mixed success of these initiatives is crucial, as it can inform future health promotion strategies and enhance their effectiveness [18].

A critical aspect of health promotion efforts is the evaluation of their impact [19]. Although various initiatives have been launched, comprehensive assessments are lacking, making it difficult to determine their overall effectiveness and utilization rates among different demographic segments. Evaluating these programs not only assists in identifying best practices but also supports the optimization of future health interventions [20].

There is no study that has examined the overall utilization and effectiveness of the launched initiatives to reduce health disparities among different demographic groups. To comprehensively examine the use and perceived effectiveness of health promotion initiatives in Saudi Arabia, deeper insights are needed into the factors that influence individuals’ use of health promotion initiatives and their perceptions of their impact on health outcomes. Thus, this study seeks to address an essential gap in the literature by examining the utilization and perceived effectiveness of health promotion initiatives among people in Saudi Arabia.

## 2. Materials and Methods

### 2.1. Study Design and Participants

A cross-sectional study design with a non-probabilistic convenience sampling technique was used among adults in Saudi Arabia. The inclusion criteria required participants to be over 18 years old and willing to participate, with no gender restrictions applied.

### 2.2. Ethical Concerns

The Institutional Review Board (IRB) at Princess Nourah bint Abdulrahman University (PNU), Riyadh, Saudi Arabia, approved the study before taking part (IRB log number: 23-0176). During the approval process, several key ethical considerations were thoroughly addressed to ensure the protection of participants’ rights. Participants’ identities were kept confidential throughout the study, using unique identification codes that were assigned to each participant, ensuring that no personal information was linked to the data collected. Also, data were stored securely, with access limited to authorized personnel only. All identifying information was removed from the datasets, and findings were reported in aggregate form to prevent the identification of individual participants.

All participants were informed about the study’s objectives and their rights, including the right to withdraw at any point without obligation. Informed consent was obtained digitally, and participants were provided with comprehensive documentation regarding their rights and the study’s aims.

### 2.3. Data Collection and Tool Validation

The data were collected through an online questionnaire in Arabic, developed specifically for this study, and distributed across the Kingdom of Saudi Arabia from 25 February to 8 March 2023. Before widespread distribution, the questionnaire was initially shared with 10 individuals to assess its readability and clarity. Feedback from these participants highlighted areas for improvement, such as simplifying complex questions and enhancing overall flow. As a result, several questions were rephrased for clarity and readability. Furthermore, two experts evaluated the questionnaire for its validity, ensuring the accuracy and relevance of the content. The evaluation focused on two key aspects: (a) content validity by assessing whether the items in the questionnaire adequately represented the constructs being measured, ensuring that all relevant topics were covered comprehensively, and (b) face validity by examining whether the items appeared appropriate and relevant for the intended population. The experts provided valuable insights and feedback, which contributed to the refinement of the questionnaire to align it with the study’s objectives better. In instances of disagreement or differing suggestions, a collaborative discussion was conducted to reach a consensus on necessary revisions. These revisions enhanced the clarity and relevance of several items, ensuring that the final version of the questionnaire effectively captures the intended data and addresses the research questions.

An English version is shown in Supplementary File S1. The questionnaire was created by the research team using Google Forms, and it was distributed through social media platforms such as WhatsApp, Twitter, Telegram, Snapchat, and Facebook.

### 2.4. Study Measures

#### 2.4.1. The Independent Variables

The potential predictors were sociodemographic factors and health status. First, the sociodemographic data consisted of age, sex, nationality, current residency, marital status, education level, type of housing, occupation status, and monthly income. These specific factors were chosen based on their relevance to the research objectives and existing literature that highlights their potential impact on health promotion initiatives. By including these sociodemographic factors, the study aims to capture a comprehensive picture of how individual characteristics influence perceptions and utilization of health promotion initiatives, ultimately facilitating targeted recommendations for improvement.

The current region of residence was categorized into five regions: central, southern, northern, eastern, and western. The central region (Najd) includes Riyadh, while the southern region features highlands and provinces like Aseer, Al-Baha, Najran, and Jizan, known for their fertile landscapes and higher rainfall [21]. The northern borders, with the lowest population density, borders Iraq and Jordan and has historical significance due to ancient ruins nearby [22]. The eastern region is home to the world’s largest oil field, Ghawar, while the western region, also called Hejaz, encompasses the holy cities of Mecca and Madinah, along with major urban centers such as Jeddah and Taif [21].

The health status data included height, weight, and the presence of any perceived or diagnosed physical or psychological illness. These factors were selected due to their significant relevance to the research objectives and their hypothesized impact on individuals’ perceptions and utilization of community health initiatives, ultimately informing the study’s recommendations.

#### 2.4.2. Outcome Measures

People were asked about their perceptions regarding the community health initiatives the authorities and ministries launched. Five initiatives were considered in this study (healthy malls, walking paths, labeled calories in restaurants’ menus, selling fresh juices without added sugar, and confiscating and Taxing selective drinks initiatives). A survey was conducted to assess the perceived effectiveness of these initiatives using a scale ranging from 1 to 4 (1 = not effective, 4 = very effective). The respondents were also asked to rate their utilization of these initiatives during the past 12 months on a scale ranging from 1 to 5 (1 = never, 5 = always).

The data were collected, checked for completeness, cleaned, and managed to maintain a high-quality dataset for analysis. We addressed missing data by employing proxy variables or imputation methods where appropriate. Outliers were identified and examined to determine whether they were valid responses or errors.

### 2.5. Statistical Analysis

The analysis was conducted using the IBM Statistical Package for Social Sciences (IBM-SPSS Inc., Chicago, IL, USA) version 28. Frequencies and percentages were calculated for the participants’ demographic characteristics and health status. Means and standard deviations were computed for participants’ utilization and perceptions of the health community initiatives. Multivariate ordinary least squares (OLS) regressions were employed to examine the associations of utilization and perceived effectiveness of health promotion initiatives with independent factors. The specific independent variables included sociodemographic factors (age, sex, nationality, current residency, marital status, education level, type of housing, occupation status, and monthly income), and health status factors were treated as the main variables within the same model. A *p*-value of less than 0.05 was considered statistically significant.

## 3. Results

### 3.1. Study Population Characteristics

Table 1 displays the demographic and socioeconomic characteristics of the participants. Of the 999 participants, 64.3% were females, 65.8% had a bachelor’s degree, and 41.1% were employed. Furthermore, 67.1% lived in their families’ houses (a housing unit where four people or more live), and 72.6% lived in the central region of Saudi Arabia. Regarding body mass index (BMI), 35.0% of participants had an average weight, while only 7.2% were underweight. The remaining 58% were overweight, obese, or extremely obese. Most participants had no perceived physical (86.2%) or psychological (85.6%) problems that could limit their daily activities. Similarly, many reported no diagnosed chronic physical (79.5%) or mental (93.3%) illnesses.

### 3.2. Perceived Effectiveness and Utilization of Health Promotion Initiatives

Table 2 displays the scores of the participants’ perceived effectiveness and utilization of the health promotion initiatives in Saudi Arabia. The scores of perceived effectiveness ranged from 2.73 ± 1.099 for tax on selective drinks to 3.25 ± 0.813 for walking paths. For the utilization of health promotion initiatives, the scores ranged from 2.26 ± 1.078 for healthy malls to 3.06 ± 1.253 for menus with labeled calories in restaurants.

### 3.3. Factors Associated with the Perceived Effectiveness of Health Promotion Initiatives

The results of multivariate analyses of the associations between the perceived effectiveness of the health promotion initiatives and the independent variables are demonstrated in Table 3. There were significant associations of perceived effectiveness related to healthy malls with age, family income, housing status, and perceived physical health. People aged 24 to 44 years had lower scores of perceived effectiveness related to healthy malls (beta = −0.90, *p* < 0.001) than those aged ≥ 50 years. Participants with a family income lower than SAR 15,000 per month had lower scores of perceived effectiveness related to healthy malls than those with a higher monthly income (beta = −0.43, *p* < 0.001). Additionally, individuals with no physical problems that could limit their daily activities had higher scores of perceived effectiveness related to healthy malls (beta = 0.69, *p* < 0.001) than those who perceived physical problems. The perceived effectiveness of walking paths was significantly associated with age and weight status. People aged 24 to 44 had lower scores (beta = −0.60, *p* < 0.001) than those aged ≥ 50 years. Participants who were overweight had higher scores of perceived effectiveness related to walking paths than those who were extremely obese (beta = 0.49, *p* < 0.001). Concerning labeled calories in the menus of restaurants, those who reported having no diagnosed physical diseases had higher scores of perceived effectiveness (beta = 0.44, *p* < 0.001) than those who had chronic physical diseases.

For tax on selective drinks, nationality and occupation were significantly associated with the perceived effectiveness of this health promotion initiative. For example, Saudi participants had lower scores for perceived effectiveness related to taxes on selective drinks (beta = −0.62, *p* < 0.001) than non-Saudi participants. Finally, factors, including gender, occupation, region of residence, and presence of chronic physical illness, were significantly associated with the perceived effectiveness of selling fresh juice without added sugar. Compared with females, males had lower scores of perceived effectiveness related to selling fresh juice without added sugar (beta = −0.41, *p* < 0.001). Participants who lived in the western region had higher scores of perceived effectiveness related to selling fresh juice without added sugar (beta = 0.83, *p* < 0.001) than those who lived in the eastern region. However, participants who reported having no chronic physical disease had lower scores of perceived effectiveness related to selling fresh juice without added sugar than those who reported having chronic disease (beta = −0.33, *p* > 0.001).

### 3.4. Factors Associated with the Utilization of Health Promotion Initiatives

The results of multivariate analyses demonstrated that the utilization of healthy malls was significantly associated with marital status, family monthly income, and perceived physical health. Compared with married participants, unmarried participants had lower scores for utilizing healthy malls (beta = −0.68, *p* < 0.001). People with incomes less than SAR 7000 had significantly greater scores for utilizing health malls than those with higher incomes (beta = 0.45, *p* < 0.001). People with no perceived physical illness had higher scores for utilizing healthy malls than those with physical illness (beta = 0.42, *p* < 0.001). Using walking paths was significantly associated with occupation, family monthly income, housing status, weight status, perceived physical health, and chronic mental diseases. Participants with incomes less than SAR 7000 had significantly greater scores for utilizing walking paths than those with higher incomes (beta = 0.47, *p* < 0.001). People who were overweight had higher scores for utilizing walking paths than those who were extremely obese (beta = 0.55, *p* < 0.001). Participants who perceived that they had no physical problems limiting their daily activities had higher scores for utilizing walking paths than those who perceived that they were physically ill (beta = 0.79, *p* < 0.001). Individuals who reported having no chronic psychiatric/mental diseases had higher scores for utilizing walking paths than those who had diseases (beta = 0.56, *p* < 0.001). Labeled calories were significantly associated with gender, age, family income, and weight status. Young participants (aged less than 24 years) had higher scores for utilizing labeled calories than older participants (beta = 0.83, *p* < 0.001). Compared with highly obese individuals, obese individuals had greater scores for using labeled calories (beta = 0.50, *p* < 0.001). For the tax on selective drinks, factors, including age, housing, region of residence, and weight status, were significantly associated with consuming soft drinks, energy drinks, and sweetened drinks. Young participants (aged less than 24 years) had higher scores for utilizing these drinks (soft drinks, energy drinks, and sweetened drinks) than older participants (beta = 1.30, *p* < 0.001). Individuals who lived in the northern region of Saudi Arabia had higher scores for utilizing soft drinks, energy drinks, and sweetened drinks than those who lived in the eastern region (beta = 1.43, *p* < 0.001). Individuals who had an average weight had lower scores for utilizing soft drinks, energy drinks, and sweetened drinks than those who were extremely obese (beta = − 0.69, *p* < 0.001). Consuming fresh juice without added sugar was significantly associated with age and occupation. Young participants had higher scores for consumption of unsweetened fresh juice than older participants (beta = 0.79, *p* < 0.001) (Table 4).

## 4. Discussion

The present study provided valuable insights into the demographic, socioeconomic, and health-related factors associated with health promotion initiatives’ perceived effectiveness and utilization in Saudi Arabia.

Nearly two-thirds of the sample predominantly consisted of females and individuals with bachelor’s degrees, and a significant proportion were employed. Most lived in family households, and about three-quarters of the study participants were from the central region of Saudi Arabia. The BMI indicated that half of the participants were overweight, obese, or extremely obese, and a lesser percentage were classified as normal weight or underweight.

For the perceived effectiveness of health promotion initiatives, participants rated the effectiveness of various initiatives differently. Outdoor walking paths were rated with the highest scores, while taxes on selective drinks (soft drinks, energy drinks, and sweetened drinks) received lower scores than others. Initiatives perceived as more beneficial (such as walking paths) may align well with community preferences, and perceived health benefits could influence their adoption of healthy lifestyles and long-term impact.

For the utilization of health promotion initiatives, labeled caloric menus were rated with the highest score, while healthy malls were rated with the lowest score. The relatively lower utilization score for walking paths in healthy malls suggested that this initiative could encounter challenges in attracting and engaging participants, such as accessibility issues, individual health needs, or competing interests inside the malls, including shopping, drinking coffee, or eating in restaurants versus physical activities.

The heterogeneity in the perceptions of effectiveness and utilization reflects the multifaceted nature of health promotion strategies and people’s preferences. For a detailed exploration of the factors influencing the perceived effectiveness and utilization of these initiatives, our analyses revealed several significant multivariate associations for each initiative. Although some initiatives could have the same community health objectives, people’s perceived effectiveness and utilization of these initiatives may be associated with different factors. The perceived effectiveness of walking paths in healthy malls was significantly associated with age, family income, housing, and physical health, while the perceived effectiveness of outdoor walking paths was significantly associated with only age and weight status. Participants aged 24 to 44 years perceived walking paths in healthy malls and outdoor walking paths to be less effective than those aged 50 years and older. However, this could suggest that younger adults might have different perceptions regarding walking paths, and this age disparity in perception had no significant association with using walking paths.

On the other hand, weight status was significantly associated with the perceived effectiveness and utilization of outdoor walking paths, while it was not significantly associated with walking paths in healthy malls. Individuals who were overweight had higher scores of perceived effectiveness and utilization of outdoor walking paths than those who were extremely obese. This result is consistent with a previous study conducted in Sumter County of South Carolina, where using trials was also associated with approximately three times greater odds of being overweight than being obese [23]. Similarly, obese participants had significantly greater scores for utilizing labeled caloric menus in restaurants than did extremely obese participants. This finding was also consistent with a large study conducted among young adults in the Minneapolis-StPaul metropolitan area of Minnesota, and multivariate analyses showed that weight status was significantly related to the use of calorie-related formation [24]. Conversely, people who were average weight and overweight were significantly less likely to consume soft drinks, energy drinks, and sweetened drinks than those who were extremely obese. Concerning gender, males were significantly less likely to perceive selling fresh juice without added sugar as an effective initiative. Following previous literature conducted in the USA, males were also less likely to use calorie-related information in restaurant menus than females in Saudi Arabia [25,26]. Unmarried individuals had significantly lower walking path scores in healthy malls than married individuals. This finding was compatible with a previous study conducted among students in Saudi Arabia that showed that married students had higher levels of physical activity than unmarried students [27]. Although the educational level was not significantly associated with the perceived effectiveness and utilization of any of the studied health promotion initiatives, the occupation had significant associations with the perceived effectiveness of tax on selective drinks and selling fresh juice without added sugar and with the utilization of walking paths and selling fresh juice without added sugar. Family income was significantly associated with the perceived effectiveness of walking only in healthy malls and using walking paths in healthy malls, outdoor walking paths, and calorie-related information in restaurant menus. Housing status was only associated with outdoor walking paths, where individuals who lived in family villas had significantly greater scores for utilizing these paths than others. For the region of residence, people living in the western region rated selling fresh juice without added sugar as an effective initiative compared to people living in the eastern region. In contrast, individuals who lived in the northern region had higher scores for consuming soft drinks, energy drinks, and sweetened drinks than those who lived in the eastern region. Perceived physical health positively impacts the perceived effectiveness of walking paths in healthy malls and the utilization of walking paths in parks and healthy malls. People who reported having no physical chronic disease had significantly higher scores of perceived effectiveness for labeled calories and lower scores of perceived effectiveness for selling fresh juice without added sugar than those who reported having chronic diseases. Thus, individuals with chronic diseases may perceive the availability of fresh juice without added sugar as a beneficial service compared to those who have no chronic diseases. In contrast, the presence of mental or psychological disease was significantly associated with the use of outdoor walking paths. People with no psychological disease had greater scores for the utilization of walking paths than those with psychological disease.

The current study has potential strengths and limitations that should be considered. This study is the first to examine people’s perceptions and utilization of the most common health promotion initiatives using a large sample size with comprehensive sociodemographic variables. Convenience sampling was chosen to facilitate broad data collection across diverse geographical locations, allowing us to access more potential respondents within a limited timeframe. While convenience sampling provides advantages in speed and accessibility, it has inherent drawbacks, including the potential for selection bias and limited representativeness of the broader population. To address these concerns, we distributed the questionnaire through various social media platforms, such as WhatsApp, Twitter, Telegram, Snapchat, and Facebook, to ensure exposure to a diverse demographic across the Kingdom. However, using social media may have introduced selection bias, as individuals without access to these platforms were excluded from participation. This limitation could affect the generalizability of our findings to the broader population of Saudi Arabia, particularly among demographics that may be less active on social media. Future studies should consider employing a more inclusive sampling strategy to capture a wider range of perspectives. Moreover, disease diagnosis, weight, and height were self-reported, which could have led to recall bias. While the participants were asked to recall their utilization over the past year, responding 1 ”never” to 5 “always,” further research should specify the frequency associated with each rating.

In summary, walking paths in healthy malls were perceived to be significantly less effective by individuals aged 25 to 44 years old and/or those with low income compared to their counterparts, while those with positive physical health perceived this initiative as effective compared to those with adverse physical health. Outdoor walking paths were also perceived to be significantly less effective by individuals aged 25 to 44 years than by those aged older, while those who were overweight perceived this initiative as effective compared to those who were extremely obese. Compared to those who perceived that they had physical health problems, individuals who perceived that they had no physical health problems had high scores for the effectiveness of calorie-related information on the menus of restaurants. Saudi individuals had lower scores for the perceived effectiveness of taxes on soft drinks than non-Saudi individuals. Males and/or participants who had no chronic physical illness had low scores of perceived effectiveness for selling fresh juice without added sugar compared to their counterparts. On the other hand, people who lived in the western region perceived this initiative as effective compared to those who lived in the eastern region. For utilization, walking paths in healthy malls were utilized more by those who were married, had an income less than SAR 7000, and had no physical problems than their counterparts. Individuals who were overweight, had an income less than SAR 7000, lived in a family house, or had no diagnosed chronic mental/psychological or physical problems, utilized outdoor walking paths more than their counterparts. Compared with those in other groups, caloric information in restaurant menus was more likely to be utilized by females, younger individuals (those younger than 24 years old), and obese individuals. Soft drinks, energy drinks, and sweetened drinks were consumed more by those who were young (less than 44 years old), were extremely obese, lived in apartments, and/or resided in the northern region. Finally, fresh juice without added sugar was more likely to be consumed at younger ages (younger than 44 years old) than at older ages (older than or equal to 55 years old).

## 5. Conclusions

Several initiatives were evaluated, but walking paths were the most effective, and the calorie-related information on the menus of restaurants was the most commonly used. Future studies are needed to investigate different age groups, such as teenagers, specific diseases rather than whether diseases are present. These findings highlight the importance of tailoring health promotion efforts to specific demographic and health-related characteristics.

## Figures and Tables

**Table 1:** Characteristics of the study sample (n = 999).

Variables	Frequency	(%)
Sex		
Male	357	35.7
Female	642	64.3
Nationality		
Saudi	953	95.4
Non-Saudi	46	4.6
Age (yrs)		
18–24	363	36.3
25–44	333	33.3
45–54	184	18.4
More than or equal to 55	119	11.9
Marital status		
Unmarried	447	44.7
Married	552	55.3
Educational level		
Pre-undergraduate	190	19.0
Undergraduate	657	65.8
Postgraduate	152	15.2
Occupation		
Housewife	136	13.6
A student in a health major	160	16.0
A student in a non-health major	141	14.1
Unemployed	151	15.1
An employee in the health field	43	4.3
An employee in a non-health field	368	36.8
Family monthly income		
Less than 7000 Saudi riyals	232	23.2
From 7000 to 15,000 Saudi riyals	301	30.1
From 15,000 to 25,000 Saudi riyals	241	24.1
More than 25,000 Saudi riyals	225	22.5
What type of housing do you live in?		
Family house	670	67.1
Duplex	70	7.0
Apartments	190	19.0
Other	69	6.9
In which region of the KSA do you live?		
Central region	725	72.6
Southern region	91	9.1
Northern region	14	1.4
Western region	88	8.8
Eastern region	81	8.1
Body Mass Index (BMI) (kg/m^2^):		
Underweight	72	7.2
Normal weight	344	34.4
Overweight	297	29.7
Obese	158	15.8
Extremely obese	79	7.9
Presence of physical problems that limit daily activities:		
Yes	138	13.8
No	861	86.2
Presence of psychological problems that limit daily activities:		
Yes	144	14.4
No	855	85.6
Presence of any diagnosed chronic physical illness:		
Yes	205	20.5
No	794	79.5
Presence of any diagnosed chronic mental illness:		
Yes	67	6.7
No	932	93.3

Data are represented as frequencies and percentages (%). KSA: Kingdom of Saudi Arabia.

**Table 2:** The Participants’ perceived effectiveness and utilization regarding the health promotion initiatives in Saudi Arabia (n = 999).

Variables	Mean	SD
The perceived effectiveness of the initiatives		
Healthy mall	2.95	0.905
Walking paths	3.25	0.813
Labeled calories menus in restaurants	3.19	0.922
Tax on selective drinks	2.73	1.099
Selling fresh juices without added sugar or confiscates	3.09	0.938
Utilization of the initiatives by participants during the last 12 months		
Healthy malls	2.26	1.078
Walking paths	2.81	1.124
Labeled calories menus in restaurants	3.06	1.253
Tax on selective drinks	2.68	1.179
Selling fresh juices without added sugar or confiscates	2.56	1.127

Data are represented as mean ± standard deviation (SD).

**Table 3:** The adjusted associations of perceived effectiveness of the health promotion initiatives with the sample’s characteristics using ordinary least squares (OLS) regression.

Variables	Healthy Mall	Walking Paths	Labeled Calories	Tax on Selective Drinks	Selling Fresh Juices Without Added Sugar
	**Beta (95% CI)**	**Beta (95% CI)**	**Beta (95% CI)**	**Beta (95% CI)**	**Beta (95% CI)**
Sex					
Male	−0.30 (−0.62, 0.02)	−0.23 (−0.56, 0.10)	−0.20 (−0.53, 0.13)	−0.16 (−0.45, 0.16)	−0.41 (−0.74, −0.09) *
Female					
Nationality					
Saudi	−0.01 (−0.56, 0.54)	0.14 (−0.46, 0.74)	0.15 (−0.45, 0.75)	−0.62 (−1.22, −0.03) *	−0.21 (−0.80, 0.39)
Non-Saudi					
Age (yrs.)					
18–24	−0.44 (−1.13, 0.23)	−0.58 (−1.29, 0.13)	0.33 (−0.38, 1.04)	0.08 (−0.59, 0.76)	−0.08 (−0.78, 0.62)
25–44	−0.90 (−1.40, −0.40) *	−0.60 (−1.11, −0.08) *	0.09 (−0.40, 0.59)	−0.32 (−0.81, 0.16)	−0.37 (−0.87, 0.13)
45–54	−0.25 (−0.75, 0.26)	0.04 (−0.49, 0.57)	−0.04 (−0.55, 0.46)	−0.06 (−0.55, 0.44)	−0.07 (−0.58, 0.45)
≥55					
Marital status					
Unmarried	−0.12 (−0.53, 0.29)	−0.38 (−0.82, 0.06)	0.13 (−0.31, 0.58)	−0.27 (−0.69, 0.16)	0.15 (−0.28, 0.59)
Married					
Educational level					
Pre-undergraduate	−0.24 (−0.70, 0.22)	0.45 (−0.05, 0.95)	0.27 (−0.22, 0.76)	0.22 (−0.26, 0.69)	0.13 (−0.36, 0.62)
Undergraduate	−0.01 (−0.39, 0.36)	0.45 (0.04, 0.85)	0.38 (−0.01, 0.78)	0.26 (−0.12, 0.65)	0.10 (−0.29, 0.50)
Postgraduate					
Occupation					
Housewife	−0.01 (−0.44, 0.44)	0.21 (−0.27, 0.69)	−0.13 (−0.59, 0.34)	−0.36 (−0.80, 0.09)	−0.64 (−0.53, 0.40)
A student in a health major	0.23 (−0.29, 0.76)	0.16 (−0.41, 0.72)	−0.01 (−0.59, 0.57)	−0.13 (−0.68, 0.42)	−0.18 (−0.75, 0.38)
A student in a non-health major	−0.10 (−0.61, 0.41)	0.15 (−0.41, 0.70)	−0.04 (−0.61, 0.53)	−0.62 (−1.16, −0.09) *	−0.66 (−1.21, −0.11) *
Unemployed	0.04 (−0.36, 0.44)	0.11 (−0.33, 0.56)	−0.24 (−0.67, 0.19)	−0.17 (−0.59, 0.25)	−0.32 (−0.75, 0.11)
An employee in the health field	0.19 (−0.39, 0.77)	−0.05 (−0.67, 0.58)	0.00 (−0.62, 0.62)	−0.92 (−1.53, −0.31) *	−0.52 (−1.13, 0.09)
An employee in a non-health field					
Family monthly income					
SAR < 7000	−0.43 (−0.81, −0.05) *	0.30 (−0.12, 0.71)	0.11 (−0.30, 0.53)	0.03 (−0.36, 0.43)	−0.14 (−0.54, 0.27)
SAR 7000 to 15,000	−0.43 (−0.77, −0.09) *	−0.06 (−0.43, 0.31)	−0.06 (−0.43, 0.31)	0.00 (−0.35, 0.35)	−0.03 (−0.39, 0.33)
SAR 15,000 to 25,000	0.52 (0.02, 1.02)	−0.19 (−0.56, 0.18)	0.09 (−0.27, 0.46)	−0.04 (−0.39, 0.31)	−0.03 (−0.39, 0.32)
>25,000 Saudi riyals					
What type of housing do you live in?					
Family house	0.65 (0.18, 1.12) *	0.17 (−0.35, 0.68)	0.08 (−0.43, 0.59)	−0.02 (−0.51, 0.47)	−0.02 (−0.50, 0.50)
Duplex	0.73 (0.12, 1.34) *	0.16 (−0.51, 0.83)	0.24 (−0.43, 0.91)	−0.02 (−0.66, 0.62)	0.50 (−0.16, 1.17)
Apartments	0.52 (0.02, 1.02) *	0.03 (−0.51, 0.58)	−0.10 (−0.64, 0.44)	−0.20 (−0.72, 0.32)	−0.07 (−0.60, 0.46)
Other					
In which region of the KSA do you live?					
Central region	0.37 (−0.06, 0.80)	0.16 (−0.30, 0.63)	0.32 (−0.14, 0.78)	0.14 (−0.31, 0.58)	0.44 (−0.02, 0.89)
Southern region	−0.26 (−0.82, 0.29)	−0.03 (−0.63, 0.53)	−0.12 (−0.71, 0.47)	0.28 (−0.30, 0.85)	−0.04 (−0.62, 0.54)
Northern region	0.25 (−0.79, 1.30)	−0.05 (−1.18, 1.07)	−0.41 (−1.51, 0.69)	−0.05 (−1.14, 1.03)	−1.06 (−2.16, 0.05)
Western region	0.12 (−0.44, 0.69)	0.20 (−0.42, 0.52)	0.57 (−0.05, 1.19)	0.51 (−0.09, 1.10)	0.83 (0.22, 1.44) *
Eastern region					
Body Mass Index (BMI) (kg/m^2^):					
Underweight	0.35 (−0.27, 0.96)	0.62 (−0.04, 1.28)	−0.16 (−0.82, 0.51)	0.48 (−0.19, 1.12)	0.36 (−0.30, 1.01)
Normal weight	0.10 (−0.36, 0.57)	0.48 (−0.02, 0.98)	0.07 (−0.43, 0.52)	0.46 (−0.02, 0.95)	0.38 (−0.12, 0.87)
Overweight	0.31 (−0.14, 0.76)	0.49 (0.01, 0.98) *	0.04 (−0.44, 0.52)	0.11 (−0.36, 0.57)	0.21 (−0.27, 0.68)
Obese	−0.23 (−0.71, 0.25)	0.47 (−0.05, 0.99)	0.32 (−0.20, 0.84)	0.33 (−0.17, 0.83)	0.43 (−0.9, 0.95)
Extremely obese					
Perceived physical problems that limit daily activities:					
No	0.69 (0.33, 1.04) *	0.05 (−0.34, 0.43)	0.08 (−0.30, 0.46)	0.07 (−0.30, 0.43)	−0.02 (−0.40, 0.36)
Yes					
Perceived psychological problems that limit daily activities:					
No	0.55 (0.18, 0.91)	0.17 (−0.23, 0.56)	−0.05 (−0.45, 0.36)	−0.11 (−0.49, 0.27)	0.10 (−0.29, 0.49)
Yes					
Presence of any diagnosed chronic physical illness:					
No	0.03 (−0.28, 0.34)	0.28 (−0.05, 0.84)	0.44 (0.11, 0.76) *	−0.04 (−0.36, 0.28)	−0.33(−0.67, −0.02) *
Yes					
Presence of any diagnosed chronic mental illness:					
No	−0.02 (−0.50, 0.45)	0.33 (−0.18, 0.84)	0.04 (−0.48, 0.56)	0.04 (−0.46, 0.53)	0.43 (−0.08, 0.93)
Yes					

* *p*-value < 0.05, 95%CI: 95% confidence interval.

**Table 4:** The adjusted associations of utilizations related to health promotion initiatives with the sample’s characteristics using ordinary least squares (OLS) regression.

Variables	Healthy Mall	Walking Paths	Labeled Calories Menus in Restaurants.	Soft Drinks, Energy Drinks, and Sweetened Drinks	Selling Fresh Juices without Added Sugar or Confiscates
	**Beta (95% CI)**	**Beta (95% CI)**	**Beta (95% CI)**	**Beta (95% CI)**	**Beta (95% CI)**
Gender					
Male	−0.02 (−0.34, 0.30)	0.18 (−0.14, 0.50)	−0.34 (−0.66, −0.03) *	0.27 (−0.04, 0.59)	0.17 (−0.15, 0.48)
Female					
Nationality					
Saudi	−0.33 (−0.91, 0.25)	−0.20 (−0.78, 0.38)	−0.01 (−0.58, 0.56)	0.50 (−0.07, 1.08)	0.09 (−0.49, 0.66)
Non-Saudi					
Age (yrs)					
18–24	0.46 (−0.23, 1.14)	−0.09 (−0.77, 0.59)	0.83 (0.16, 1.50) *	1.30 (0.62, 1.98) *	0.79 (0.11, 1.47) *
25–44	−0.37 (−0.86, 0.12)	−0.19 (−0.68, 0.29)	0.21 (−0.27, 0.69)	1.08 (0.58, 1.57) *	0.68 (0.19, 1.17) *
45–54	0.17 (−0.33, 0.66)	0.16 (−0.34, 0.65)	0.19 (−0.30, 0.68)	0.47 (−0.02, 0.97)	0.46 (−0.03, 0.96)
≥55					
Marital status					
Non-married	−0.68 (−1.11, −0.24) *	−0.15 (−0.57, 0.28)	−0.14 (−0.56, 0.28)	0.15 (−0.28, 0.58)	−0.37 (−0.79, 0.06)
Married					
Educational level					
Pre-undergraduate	−0.01 (−0.49, 0.47)	−0.05 (−0.53, 0.42)	−0.05 (−0.52, 0.42)	0.03 (−0.45, 0.50)	0.36 (−0.12, 0.83)
Undergraduate	−0.17 (−0.56, 0.23)	−0.12 (−0.51, 0.27)	−0.11 (−0.50, 0.27)	−0.09 (−0.48, 0.30)	0.14 (−0.24, 0.53)
Postgraduate					
Occupation					
Housewife	−0.12 (−0.58, 0.33)	−0.17 (−0.62, 0.28)	−0.29 (−0.73, 0.15)	−0.07 (−0.52, 0.37)	−0.59 (−1.04, −0.14) *
A student in a health major	−0.16 (−0.71, 0.40)	−0.57 (−1.11, −0.02) *	0.13 (−0.41, 0.67)	0.24 (−0.31, 0.78)	0.07 (−0.25, 0.53)
A student in a non-health major	0.15 (−0.39, 0.68)	−0.26 (−0.79, 0.28)	0.28 (−0.27, 0.81)	0.07 (−0.46, 0.60)	0.20 (−0.33, 0.73)
Unemployed	0.24 (−0.18, 0.67)	0.02 (−0.40, 0.44)	0.14 (−0.28, 0.56)	−0.16 (−0.58, 0.26)	−0.21 (−0.63, 0.21)
An employee in the health field	−0.05 (−0.67, 0.56)	−0.30 (−0.90, 0.31)	−0.01 (−0.61, 0.59)	0.07 (−0.53, 0.67)	−0.21 (−0.82, 0.39)
An employee in a non-health field					
Family monthly income					
SAR < 7000	0.45 (0.06, 0.85) *	0.47 (0.08, 0.86) *	−0.24 (−0.63, 0.15)	0.08 (−0.32, 0.47)	0.37 (−0.01, 0.78)
SAR 7000 to 15,000	0.16 (−0.20, 0.50)	0.01 (−0.34, 0.37)	−0.36 (−0.71, −0.01) *	−0.09 (−0.44, 0.26)	0.22 (−0.13, 0.57)
SAR 15,000 to 25,000	0.15 (−0.20, 0.50)	0.10 (−0.25, 0.45)	−0.11 (−0.46, 0.24)	−0.34 (−0.69, 0.01)	−0.07 (−0.42, 0.28)
>25,000 Saudi riyals					
What type of housing do you live in?					
Family house	0.26 (−0.24, 0.76)	0.57 (0.08, 1.06) *	0.17 (0.32. 0.65)	0.33 (−0.16, 0.82)	0.11 (−0.38, 0.59)
Duplex	0.23 (−0.41, 0.87)	0.25 (−0.39, 0.89)	0.52 (−0.11, 1.15)	0.40 (−0.23, 1.03)	−0.01 (−0.63, 0.63)
Apartments	0.04 (−0.49, 0.57)	0.39 (−0.14, 0.91)	0.19 (−0.32, 0.71)	0.70 (0.17, 1.22) *	−0.07 (−0.59, 0.45)
Other					
In which region of the KSA do you live?					
Central region	−0.02 (−0.47, 0.43)	−0.14 (−0.59, 0.31)	−0.18 (−0.62, 0.26)	0.10 (−0.34, 0.54)	−0.30 (−0.74, 0.15)
Southern region	−0.35 (−0.93, 0.23)	−0.22 (−0.79, 0.36)	−0.30 (−0.87, 0.27)	0.09 (−0.48, 0.66)	0.12 (−0.45, 0.70)
Northern region	−0.07 (−1.16, 1.02)	−0.12 (−1.21, 0.97)	0.23 (−0.85, 1.31)	1.43 (0.34, 2.52) *	0.57 (−0.52, 1.65)
Western region	−0.23 (−0.82, 0.37)	−0.22 (−0.81, 0.37)	−0.30 (−0.88, 0.29)	−0.29 (−0.88, 0.30)	−0.42 (−1.01, 0.17)
Eastern region					
Body Mass Index (BMI) (kg/m^2^):					
Underweight	−0.23 (−0.87, 0.42)	0.33 (−0.31, 0.97)	−0.09 (−0.73, 0.54)	−0.59 (−1.22, 0.05)	0.24 (−0.39, 0.88)
Normal weight	0.18 (−0.31, 0.66)	0.20 (−0.28, 0.69)	−0.13 (−0.61, 0.35)	−0.69 (−1.17, −0.21) *	−0.13 (−0.61, 0.35)
Overweight	0.20 (−0.28, 0.67)	0.55 (0.08, 1.02)*	0.28 (−0.18, 0.75)	−0.58 (−1.05, −0.11) *	−0.09 (−0.56, 0.38)
Obese	0.12 (−0.39, 0.63)	0.04 (−0.46, 0.54)	0.50 (0.00, 1.00) *	−0.27 (−0.77, 0.27)	−0.33 (−0.83, 0.17)
Extremely obese					
Perceived physical problems that limit daily activities:					
No	0.42 (0.05, 0.79) *	0.79 (0.53, 1.16) *	0.24 (−0.12, 0.60)	−0.15 (−0.51, 0.22)	−0.32 (−0.68, 0.05)
Yes					
Perceived psychological problems that limit daily activities:					
No	0.03 (−0.36, 0.41)	−0.12 (−0.50, 0.26)	−0.03 (−0.40, 0.35)	−0.06 (−0.43, 0.32)	−0.29 (−0.67, 0.08)
Yes					
Presence of any diagnosed chronic physical illness:					
No	0.12 (−0.21, 0.44)	0.09 (−0.24, 0.41)	0.19 (−0.13, 50)	−0.02 (−0.34, 0.30)	0.18 (−0.14, 0.50)
Yes					
Presence of any diagnosed chronic mental illness:					
No	0.33 (−0.18, 0.84)	0.56 (0.06, 1.06) *	0.18 (−0.32, 0.67)	−0.03 (−0.52, 0.46)	0.18 (−0.32, 0.67)
Yes					

* *p*-value < 0.05, 95%CI: 95% confidence interval.

## Data Availability

The original contributions presented in the study are included in the article/Supplementary Material; further inquiries can be directed to the corresponding authors.

## Supplementary Materials

The following supporting information can be downloaded at https://www.mdpi.com/article/10.3390/healthcare12232352/s1, Supplementary File S1: The questionnaire.

## References

[1] Nutbeam D. (1998). Evaluating health promotion: Progress, problems and solutions. Health Promot. Int..

[2] Green L. (1991). Health Promotion Planning: An Educational and Environmental Approach.

[3] Nutbeam D. (2008). The evolving concept of health literacy. Soc. Sci. Med..

[4] World Health Organization (2021). Health Promotion Glossary of Terms 2021.

[5] Nutbeam D., Muscat D.M. (2021). Health promotion glossary 2021. Health Promot. Int..

[6] Al-Qahtani M.F. (2015). Health-promoting lifestyle behaviors among nurses in private hospitals in Al-Khobar, Saudi Arabia. J. Egypt. Public Health Assoc..

[7] Bahijri S.M., Jambi H.A., Al Raddadi R.M., Ferns G., Tuomilehto J. (2016). The Prevalence of Diabetes and Prediabetes in the Adult Population of Jeddah, Saudi ArabiaA Community-Based Survey. PLoS ONE.

[8] Alshuwaikhat H.M., Mohammed I. (2017). Sustainability Matters in National Development Visions—Evidence from Saudi Arabia’s Vision for 2030. Sustainability.

[9] AlMarzooqi M.A., Alsukait R.F., Aljuraiban G.S., Alothman S.A., AlAhmed R., Rakic S., Herbst C.H., Al-Hazzaa H.M., Alqahtani S.A. (2023). Comprehensive assessment of physical activity policies and initiatives in Saudi Arabia 2016–2022. Front. Public Health.

[10] Al-Hazzaa H.M., AlMarzooqi M.A. (2018). Descriptive Analysis of Physical Activity Initiatives for Health Promotion in Saudi Arabia. Front. Public Health.

[11] Alassaf H.I., Alaskar Y.A., Alqulaysh B.F., Alshehri M.A., Alnosian M.Y., Alshamrani A.A., Al-Tannir M.A. (2020). Assessment of knowledge, attitudes and practices of Saudi adults about calorie labeling in central Saudi Arabia. Saudi Med. J..

[12] AlAmer N.A., AlOmar R.S., AlKaltham S.M., AlYami R.S., AlRashidi F.N., AlJrri M.M., Abdel Wahab M.M. (2020). Perceived Effect of Calorie Count Display on Customers’ Eating Behaviors in Food Facilities of Eastern Province, Saudi Arabia: A Mixed Method Study. J. Multidiscip. Healthc..

[13] Chaloupka F.J., Powell L.M., Warner K.E. (2019). The Use of Excise Taxes to Reduce Tobacco, Alcohol, and Sugary Beverage Consumption. Annu. Rev. Public Health.

[14] Cabrera Escobar M.A., Veerman J.L., Tollman S.M., Bertram M.Y., Hofman K.J. (2013). Evidence that a tax on sugar sweetened beverages reduces the obesity rate: A meta-analysis. BMC Public Health.

[15] Alsukait R., Wilde P., Bleich S., Singh G., Folta S. (2019). Impact of Saudi Arabia’s Sugary Drink Tax on Prices and Purchases (P10-066-19). Curr. Dev. Nutr..

[16] AlShehri N.M., AlMarzooqi M.A. (2022). Consumers’ Knowledge, Attitudes, and Practices Toward Calorie Labeling in Riyadh City, Saudi Arabia: A Cross-Sectional Assessment. Front. Public Health.

[17] Alsukait R., Bleich S., Wilde P., Singh G., Folta S. (2020). Sugary drink excise tax policy process and implementation: Case study from Saudi Arabia. Food Policy.

[18] Barry M.M. (2021). Transformative health promotion: What is needed to advance progress?. Glob. Health Promot..

[19] Liao C.H., Bercea S. (2021). Success factors of health promotion: Evaluation by DEMATEL and M-DEMATEL methods—A case study in a non-profit organization. PLoS ONE.

[20] Gosadi I.M. (2019). National screening programs in Saudi Arabia: Overview, outcomes, and effectiveness. J. Infect. Public Health.

[21] Bowen W.H. (2024). The History of Saudi Arabia.

[22] Alenzi E.O., Fatima W., Amara A., Imran M., Shah S.S.H., Elbilgahy A.A., Fawzy M.S., Abu-Negm L.M., Mujtaba M.A., Jacinto-Caspillo I. (2023). A Systematic Review of Chronic Diseases and Their Prevalence Among the Population of Northern Borders Province (NBP) in Saudi Arabia. J. Multidiscip. Healthc..

[23] Wilson D.K., Ainsworth B.E., Bowles H. (2007). Body mass index and environmental supports for physical activity among active and inactive residents of a U. S. Southeastern county. Health Psychol..

[24] Larson N., Haynos A.F., Roberto C.A., Loth K.A., Neumark-Sztainer D. (2018). Calorie Labels on the Restaurant Menu: Is the Use of Weight-Control Behaviors Related to Ordering Decisions?. J. Acad. Nutr. Diet..

[25] Glanz K., Basil M., Maibach E., Goldberg J., Snyder D. (1998). Why Americans eat what they do: Taste, nutrition, cost, convenience, and weight control concerns as influences on food consumption. J. Am. Diet. Assoc..

[26] Ellison B., Lusk J.L., Davis D. (2013). Looking at the label and beyond: The effects of calorie labels, health consciousness, and demographics on caloric intake in restaurants. Int. J. Behav. Nutr. Phys. Act..

[27] Khalaf A., Ekblom Ö., Kowalski J., Berggren V., Westergren A., Al-Hazzaa H. (2013). Female university students’ physical activity levels and associated factors—A cross-sectional study in southwestern Saudi Arabia. Int. J. Environ. Res. Public Health.

