# Supplementary material for: Perceived Effectiveness and Utilization of Health Promotion Initiatives in Saudi Arabia: Insights and Recommendations
Source: Healthcare (Basel). 2024 Nov 25;12(23):2352. doi: 10.3390/healthcare12232352 (PMC11641317; doi:10.3390/healthcare12232352)
Supplement: Supplementary file 1 [file healthcare-12-02352-s001.zip › healthcare-3222140-supplementary.pdf]

Thank you for your interest in participating.
Please answer all the questions in the following survey :
Would you like to participate in the research ?
o Yes
o No
First: Demographic and socioeconomic characteristics:
1- What is your age?
....
2- Gender?
o Male
o Female
3- Nationality?
o Saudi
o non-Saudi
4- Marital status?
o Single
o Married
o Divorced or Widowed
5- Educational level?
o Primary
o Middle School
o High School
o University
o Postgraduate (Master's / Doctorate)
6- Occupation:
o Student in a health-related field
o Student in a non-health-related field
o Employee in the health sector
o Employee in a non-health sector
o Unemployed
o Retired
o Homemaker

7- Please select the monthly family income:
o Less than 3,000 Saudi Riyals
o 3,000 to 5,000 Saudi Riyals
o More than 5,000 to 7,000 Saudi Riyals
o More than 7,000 to 10,000 Saudi Riyals
o More than 10,000 to 15,000 Saudi Riyals
o More than 15,000 to 20,000 Saudi Riyals
o More than 20,000 to 25,000 Saudi Riyals
o More than 25,000 to 30,000 Saudi Riyals
o More than 30,000 Saudi Riyals

8- What type of residence do you live in?
o Family villa
o Duplex villa
o Shared accommodation
o Traditional Arab-style house
o Apartment with fewer than 6 units
o Apartment with more than 6 units
o Other

9-How many family members are there?
....

10-In which region of the Kingdom do you live?
o Riyadh Region
o Makkah Region
o Madinah Region
o Qassim Region
o Eastern Province
o Asir Region
o Tabuk Region
o Ha'il Region
o Northern Borders Region
o Jazan Region
o Al-Baha Region
o Al-Jouf Region

Second: Physical and Psychological Health:
1- What is your height in centimeters (cm)?
2- What is your weight in kilograms (kg)?
3- Do you suffer from any physical problem that limits your activity and daily tasks?
o Yes
o No
4- Do you suffer from any psychological problem that limits your activity and daily tasks?
o Yes
o No
5- Have you been diagnosed by a doctor with any chronic physical illness (such as diabetes or asthma)?
o Yes... Please specify
o No
6- Have you been diagnosed by a doctor with any chronic psychological illness (such as depression or anxiety)?
o Yes... Please specify
o No
Third: Understanding people's perceptions of the initiatives launched by Saudi authorities and ministries to promote health in the Kingdom of Saudi Arabia and assessing the extent to which people use them. Among these initiatives are:
1A-The initiative launched by the Ministry of Health to activate the "Healthy Mall" by allocating walking paths in several commercial centers and malls in Saudi Arabia. How do you view the effectiveness of this initiative on health in the Kingdom of Saudi Arabia?
o Very effective
o Effective
o Somewhat effective
o Not effective at all
1B-How frequently have you used these walking paths in commercial centers and malls in Saudi Arabia to practice walking (in the past year)?

o Never
o Rarely
o Sometimes
o Often
o Always

2A-The initiative launched by the Ministry of Health to allocate health-prepared walking paths in public parks available in various regions of the Kingdom. How do you view the effectiveness of this initiative on health in the Kingdom of Saudi Arabia?

o Very effective
o Effective
o Somewhat effective
o Not effective at all

2B-How frequently have you used the health-prepared walking paths in public parks available in various regions of the Kingdom (in the past year)?

o Never
o Rarely
o Sometimes
o Often
o Always

3A-The initiative launched by the Saudi Food and Drug Authority to list calorie counts on restaurant and café menus, in collaboration with the Ministry of Municipal and Rural Affairs and the Consumer Protection Association. How do you view the effectiveness of this initiative on health in the Kingdom of Saudi Arabia?

o Very effective
o Effective
o Somewhat effective
o Not effective at all

3B-How frequently have you used this initiative when ordering meals at restaurants or cafes (in the past year)?

o Never
o Rarely
o Sometimes
o Often

o Always
4A-The initiative launched by the Zakat, Tax and Customs Authority to impose a selective tax on beverages that have negative health effects, such as soft drinks, energy drinks, and sweetened drinks. How do you view the effectiveness of this initiative on health in the Kingdom of Saudi Arabia?
o Very effective
o Effective
o Somewhat effective
o Not effective at all
4B-How frequently have you consumed soft drinks, energy drinks, and sweetened beverages (in the past year)?
o Never
o Rarely
o Sometimes
o Often
o Always
5A-The initiative launched by the Saudi Food and Drug Authority requiring fresh juice vendors to avoid adding sugar or its sources (honey, glucose syrup), flavors, and colorants to fresh juices and their blends. How do you view the effectiveness of this initiative on health in the Kingdom of Saudi Arabia?
o Very effective
o Effective
o Somewhat effective
o Not effective at all
5B-How frequently have you consumed unsweetened fresh juices (in the past year)?
o Never
o Rarely
o Sometimes
o Often
o Always
